# The involvement of the primo vascular system in local enteritis and its modification by electroacupuncture

**DOI:** 10.3389/fimmu.2022.1072996

**Published:** 2023-01-11

**Authors:** Sha Nan, Juan Wan, Qianghui Lei, Xinya Wang, Ning Ma, Ruiling Yin, Jiandi Zhu, Mingxing Ding, Yi Ding

**Affiliations:** ^1^ College of Veterinary Medicine, Huazhong Agricultural University, Wuhan, China; ^2^ Gannan Innovation and Transformation Medical Research Institute, First Affiliated Hospital, Gannan Medical University, Ganzhou, China

**Keywords:** primo vascular system, local enteritis, proteomics, electroacupuncture, rabbits

## Abstract

**Introduction:**

The primo vascular system (PVS), an intensive network structure, has been claimed to be representative of the acupuncture meridian. Here, we explored the role of the PVS in local enteritis and its modification by acupuncture.

**Methods:**

Chronic cecitis in rabbits was induced by 2,4,6-trinitro-benzene-sulfonic acid (TNBS). The PVS on the cecum was visualized with trypan blue staining, and collected with the help of microsurgical forceps under an optical stereomicroscope.

**Results:**

The increased primo vessels (PVs) and primo nodes (PNs) of the PVS on the surface of the cecum were induced by local inflammation, which was positively correlated with the inflammatory cells in the cecal mucosa. Tandem mass tag (TMT) based proteomic analysis revealed that 110 differentiated proteins of the PVS existed between TNBS-treated and control rabbits; 65 proteins were upregulated, while 45 proteins were downregulated. These proteins were mainly enriched in inflammation- and immunity-related processes, such as inflammatory cell proliferation, antigen presentation, and cell adhesion in the proliferated PVS (data are available *via* ProteomeXchange with the identifiers PXD034280). Importantly, TNBS-induced cecitis, the proliferated PVS and inflammation response-related proteins (CD40, CD45, HLA-DRA1, LAMP1, JAGN1 and FGL1) in the PVS were alleviated or reversed by repetitive electroacupuncture (EA) stimulations.

**Conclusion:**

These results suggest that the proliferated PVS and its active inclusions were related to the inflammatory process, which was modified by EA. Our study provides a new avenue for further exploration of the mechanism by which EA exerts anti-inflammatory effects.

## Introduction

As early as 1962, Bonghan Kim, a scientist in North Korea, proposed an intensive vascular structure independent of blood vessels and lymphatic vessels that corresponded to the ancient acupuncture meridians (1). He successively reported this novel network structure in terms of the structural architecture, cell composition, distribution, and physical property, and named it the Bonghan system (BHS) (1–3). However, due to the lack of suitable methodological descriptions, his findings were rarely reproduced by contemporary scientists during his time. It was not until 2004 that the scientific group of Dr. Kwang-Sup Soh initiated a series of experiments to visualize BHS with multiple staining methods, and verified most of Kim’s hypotheses along with other scientists (4–6). There is increasing evidence that BHS develops earlier than the blood or nerve system; hence, it has been renamed the primo vascular system (PVS) (7). In the past 20 years, studies have verified that the PVS is widely distributed throughout the body, including inside or outside blood vessels, lymphatic vessels, subcutaneous tissue, internal organs, and the nervous system (8–13). With a specially-designed high-resolution microscope, Vodyanoy et al. observed that the PVS consists of nodular primo nodes (PNs) and interconnected primo vessels (PVs), in which translucent fluid is flowing (14, 15). Kwon et al. (10) verified an abundance of mast cells, granulocytes, histiocytes, and stem cells residing in the PNs. From its resident cells and extensive distribution, it is believed to play a crucial role in the processes of inflammation, immunity, and damage repair. However, these speculated functions of the PVS have rarely been verified.

Electroacupuncture (EA), as a non-invasive method, has been proven efficient in ameliorating various diseases and complications, including inflammatory bowel disease (IBD) and its companying visceral hypersensitivity, by stimulating specific acupoints (16–19). Many lines of evidence indicate that EA exhibits remarkable anti-inflammatory effect in various diseases (19–21). Our previously studies demonstrated that EA stimulation at ST36 inhibited 2,4,6-trinitro-benzene-sulfonic acid (TNBS)-induced inflammation *via* multiple signaling pathways in the central nerve system (16, 22). Recently, numerous studies have shown that EA at bilateral hindlimb ST36 acupoints exhibited good effects in alleviating somatic and visceral inflammation (23–25). The anti-inflammatory effect of EA is associated with alternations in the populations and functions of inflammatory cells (26–28). According to the theory of traditional Chinese medicine, EA is believed to regulate the functions of internal organs *via* the meridian system. Lim et al. (9) stained the PVS in the corium or subcutaneous tissue of the skin with Hemacolor, and found that the PVS was distributed along meridian lines. Han et al. (29) intratesticularly injected chromium hematoxylin and found the PVS distributed on the surface of the greater omentum, small intestine, and urinary bladder, indicating that PVs with different distributions are closely interconnected. These studies suggest that some association probably exists between the PVS and the acupuncture signal transmission route. Therefore, the role of the PVS in EA effects in a specific pathological state deserves investigation.

TNBS is commonly used to induce chronic bowel inflammation and hypersensitivity, and to study their underlying mechanisms. Here, local cecitis in rabbits was induced with TNBS, and the PVS on the surface of the cecum was harvested under a stereomicroscope. Differential proteins in the PVS were determined with Tandem mass tag (TMT)-based proteomic analysis. Subsequently, EA was applied to stimulate bilateral ST36 points to determine alternation in the PVS and its differential proteins under the cecitis. This study demonstrated the role of PVS in local enteritis and its regulation by EA.

## Materials and methods

### Animals

Adult male New Zealand white rabbits of 2.4 ± 0.2 kg body weight were purchased from the laboratory animal center of Huazhong Agricultural University. All rabbits were maintained in a 12 h dark/light cycle with food and water *ad libitum*. Before the experiment, they were allowed to adapt to the environment for a week. The rabbits were randomly divided into four groups: control (n = 18), TNBS-treated (n = 24), Sham-EA (n = 6), and EA (n = 6). The study was conducted under the guidelines approved by the Institutional Animal Care and Use Committee of Huazhong Agricultural University (HZAURAB-2022-0002), Wuhan, China.

### TNBS-induced cecitis

**Figure 1:**
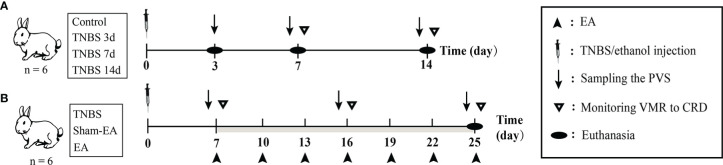
The scheme of experiment. **(A)** The diagram for 2,4,6-trinitro-benzene-sulfonic acid (TNBS)-induced cecitis. **(B)** The diagram for electroacupuncture (EA) stimulation after TNBS-induced cecitis.

Cecitis was induced after referring to previous studies (30). After being weighted, the rabbits were lightly anesthetized using 3% pentobarbital (1 mL/kg IV). To achieve moderate anesthesia, an additional 25% of the initial pentobarbital dose was injected intravenously into the rabbits. For the rabbits in the TNBS-treated, Sham-EA, and EA groups, 2 mL of 2.5% (w/v) TNBS (Sigma, USA) within 40% ethanol was slowly injected into the intestinal cavity 10 cm away from the end of the cecum, and the control group was treated with 40% ethanol. After surgery, penicillin sodium was intramuscularly administered to avoid infection for three days. Body weights of rabbits were recorded after modeling TNBS-induced cecitis on days 0, 7, and 14 (**Figure 1A**).

### Visceromotor response to colorectal distension

Visceromotor responses (VMRs) to different colorectal distension (CRD) pressures were monitored to determine the visceral hypersensitivity (VH) of rabbits according to a previous study (31). Briefly, two nickel-steel needles (0.30 mm × 13 mm) were obliquely inserted 2 cm apart into the left abdominal muscles of rabbits, and connected to an electromyography (EMG) recorder (Nanjing Medease Science and Technology Co., Ltd, China) *via* two electrodes. The polyethylene balloon was self-made and inserted into the distal cecum 8 cm from the anus of the rabbits after lubricating with paraffin oil. The pressure in the balloon monitored by a sphygmomanometer was increased from 20 to 40, 60, 80, and 100 mmHg by stage (each stage lasted for 6 s). Simultaneously, EMG data were obtained through a recorder connected to the abdominal muscles of rabbits. The areas under curve at different stages were calculated from EMG data using MedLabV6.3 software (Nanjing medease Science and Technology Co., Ltd., China), which was considered as VMRs to CRD. For the rabbits in the TNBS-treated and the control groups, the VMR to CRD was monitored on days 7 and 14. For the rabbits in the Sham-EA and EA groups, the VH was determined on days 7, 16, and 25.

### Assessment of cecal inflammation

The rabbits in the TNBS-treated group and the control groups on days 3, 7, and 14 and in the TNBS-treated, Sham-EA, and EA groups on day 25 (n = 6 per group) were sacrificed with an overdose of sodium pentobarbital to assess the inflammatory status of the cecum. The cecum specimens were collected and performed following our previous report for hematoxylin and eosin (H&E) staining and enzyme-linked immunosorbent assay (ELISA) (22). The macroscopic and microscopic changes of rabbit cecum were scored by two independent observers according to the previous studies (**Tables S1-2**) (32, 33). The number of inflammatory cells in the cecal mucosa was analyzed using the Image-Pro Plus 6.0 software (Media Cybernetics, Inc., USA). The concentrations of tumor necrosis factor α (TNF-α), interleukin 6 (IL-6), and myeloperoxidase (MPO) were measured according to the manufacturer’s instructions (Mlbio, China).

### Identification of the PVS

To identify the PVS *in vivo*, all operations were performed according to previous studies (8, 34) using an SM-2000 L binocular operative microscope (Shanghai Yide Medical Treatment Equipment Co., Ltd., China). First, an incision along the linea alba was made to open the abdominal cavity, and bleeding was controlled over time. The intestines were exposed, and a 0.4% trypan blue solution was added to the surface of the cecum for PVS staining. The tissues suspected to be PVS were specifically colored and collected with the help of microsurgical forceps. A heating pad was used for heat preservation, and warm saline was continuously dropped onto the surface of the intestine to avoid drying during the operation.

The suspected PVS was then identified based on the unique characteristics of PV: (1) rod-shaped nuclei of PV and (2) multiple sub-PVs in a single PV (2, 15). The suspected PVS samples were divided into two parts. The anterior part was fixed in 4% paraformaldehyde, embedded in Tissue-Tek O.C.T. (Sakura, Japan), sectioned in a cryostat (15 µm), and mounted on polylysine-coated slides. The slides were air-dried for 30 min at room temperature and washed with phosphate buffered saline for 3 times before staining. For diamidino-phenyl-indole (DAPI) staining, the slides were stained with DAPI (diluted 1:5000, Beyotime, China) for 10 min and observed with an EVOS M5000 fluorescence microscope (Invitrogen, USA). For H&E staining, the slides were processed according to the instructions of the H&E Staining Kit (Beyotime, China), and the nuclei of the PVs were observed with an Eclipse 80I light microscope (Nikon, Japan). After the unique nucleus shape of the PVs was determined, the posterior part of the suspected PVS was taken out for scanning electron microscopy (SEM) analysis.

The SEM analysis was performed as reported in a previous study (35). Briefly, after fixation with 2.5% glutaraldehyde in phosphate buffer at 4°C overnight, the suspected PVS samples with rod-shaped nuclei were dehydrated in ethanol, and critical point-dried. After being coated with gold, the sub-PVs of the suspected PVS samples were observed with a JSM-6390LV (JEOL, Japan) scanning electron microscope.

### TMT-based quantitative proteomic analysis of PVS

To characterize protein alterations in the PVS with inflammation, TMT-based microproteomics were performed to analyze the PVS in rabbits of the TNBS-treated and Control group. Each group contains three biological replicates. The PVS samples collected from the surface of the cecum were identified by nucleus-specific staining and SEM analysis based on the unique characteristics of PV, and then verified the PVS samples were frozen in liquid nitrogen. The proteins were extracted from the frozen PVS and separated on a 12% SDS-PAGE gel. The proteins were digested into peptides with filter-aided sample preparation (36). Each aliquot of 300 μg of PVS proteins was diluted to 200 μL with UA solution (8 M urea in 0.1 M Tris-HCl, pH 8.5) and then centrifuged in a 30-kDa filter. A UA solution with 20 mM DTT was added, and the reduction reaction was kept for 4 h at 37°C. Then, a UA solution with 50 mM iodoacetamide was added. The sample was incubated in the dark for 30 min at room temperature. After that, the ultra-fraction tube was washed with 200 μL of UA solution and 200 μL of TEAB solution (100 mM tetraethylammonium bromide) by centrifugation. Then, 100 μL of TEAB solution containing 5 μg trypsin was added to each filter tube. The tubes were incubated at 37°C for 12 h. The peptides were collected by centrifugation at 14,000g for 15 min. After measuring the concentrations, the peptides were labelled following the instructions of the TMT-10-plex Isobaric Mass Tagging Kit (Thermo, USA) (37). Subsequently, the labeled peptides were fractionated using high-pH reversed-phase chromatography and each of the fractions was analyzed by Q Exactive HF liquid chromatography tandem mass spectrometry (LC-MS/MS) (Thermo Fisher, USA) according to a previous study (38). For LC-MS/MS analysis, 0.5 μg of peptides mixture resolved in buffer A (1% formic acid (FA)) were loaded onto a 2-cm self-packed trap column (150-μm inner diameter, 1.9-μm particle size, 12-cm length) with buffer A and separated over a 78-min gradient (buffer A, 1% FA in water; buffer B, 1% FA in acetonitrile) at a flow rate of 600 nL/min (0–16 min, 6–12% B; 16–51 min, 12–24% B; 51–71 min, 24–32% B; 71–72 min, 32–95% B; and 72–78 min, 95% B). Proteins identification was performed with MaxQuant tools (version 1.5.3.8; database uniprot_oryctolagus_20180613; Matrix Science, UK). Trypsin was selected as the proteolytic enzyme. The false discovery rates of the peptide-spectrum matches and proteins were set to less than 1%. For the comparison (TNBS-treated vs. control), the fold change of discovery proteomes (*P* < 0.05 and fold change > 1.2) was set as the threshold to identify differentially expressed proteins (**Data sheet 1**). Gene ontology (GO) enrichment analysis and Kyoto Encyclopedia of Genes and Genomes (KEGG) analysis were carried out using the UniProt database (https://www.uniprot.org/). The protein–protein interaction was analyzed based on the STRING database (https://cn.string-db.org/).

### Electroacupuncture

The rabbits were given EA stimulation as previously described (16, 39). For the rabbits in the EA group, a pair of stainless-steel acupuncture needles (0.30 mm D×13 mm L, Suzhou Medical Supplies, Co., Ltd., China) was inserted 1 cm deeper at the bilateral “Zusanli” (ST-36 in humans) acupoints in hind legs. EA stimulation was applied to rabbits with a frequency of 2/100 Hz *via* stainless-steel needles connected to WQ-6F Electronic Acupuncto-scope (Beijing Xindonghua Electronic Instrument Co., Ltd., China) for 30 min. The intensity of the EA stimulation was initially set at 1 mA and increased by 1 mA every 10 min. Rabbits in the Sham-EA group received a minimal acupuncture intervention in which acupuncture needles were kept at the same acupoints without electrical stimulation or further manipulation (lifting, inserting, or twisting). The EA treatment started on day 7 after TNBS-induced cecitis, and then every three days for a total of seven times (**Figure 1B**).

### Western blotting analysis

For western blot analysis, the frozen PVS samples were grinded and lysed with radio immunoprecipitation assay lysis buffer (Beijing Solarbio Science & Technology Co., Ltd., China). Twenty micrograms of the total proteins were subjected to 10% SDS-PAGE gel and transferred to polyvinylidene fluoride membranes (Millipore; Bedford, MA). After being incubated in Tris-buffered saline containing 0.1% Tween-20 (TBST) with 5% nonfat milk for 1 h, the membranes were infiltrated with anti-tubulin-α antibody (Proteintech 66031-1-Ig, diluted 1:3000), anti-tumor necrosis factor receptor superfamily member 5 (CD40) antibody (Abclonal A0218, diluted 1:1000), anti-receptor-type tyrosine-protein phosphatase C (CD45) (Huabio ER1901-29, diluted 1:1000), anti-HLA (RLA) class II histocompatibility antigen DR alpha chain (HLA-DRA1) antibody (Abclonal A10863, diluted 1:1000), anti-TAP binding protein (TAPBP) antibody (Abclonal 13357, diluted 1:1000) anti-toll-like receptor 2 (TLR2) antibody (Abclonal A2545, diluted 1:1000), anti-semaphorin 4D (SEMA4D) antibody (Abclonal A10136, diluted 1:1000), anti-fibrinogen like 1 (FGL1) antibody (Abclonal A20335, diluted 1:1000), anti-jagunal homolog 1 (JAGN1) antibody (Santa Cruz sc-515306, diluted 1:1000), and anti-lysosomal associated membrane protein 1 (LAMP1) antibody (CST 9091, diluted 1:1000), respectively, overnight at 4°C. Subsequently, the membranes were hatched with horseradish peroxidase-conjugated secondary antibodies for 1 h, visualized with Fusion Solo S (Vilber, France), and analyzed using the Image-Pro Plus 6.0 software (Media Cybernetics, Inc., USA).

### Statistical analysis

All data were expressed as mean ± standard deviation (SD). Statistical analysis was performed using GraphPad Prism 6 software (Media Cybernetics, Inc., USA). The Mann–Whitney U-test (unpaired) was used to analyze the inflammatory scores of the cecum and the number of PVS. A one-way analysis of repeated variance, followed by Bonferroni’s *post hoc* test, was performed to assess the expression levels of TNF-α, IL-6, and MPO, as well as the changes in VMR to CRD between the different groups. An unpaired Student’s t test was used to compared the expression in the differential proteins of the PVS between the control and TNBS groups. Spearman correlation was applied to analyze the relevance between the number of PVS on the surface of the cecum and the number of inflammatory cells in the cecal mucosa using Statistical Product Service Solutions 21.0 software (SPSS Inc., USA).

## Results

### TNBS-induced cecitis in rabbits

**Figure 2:**
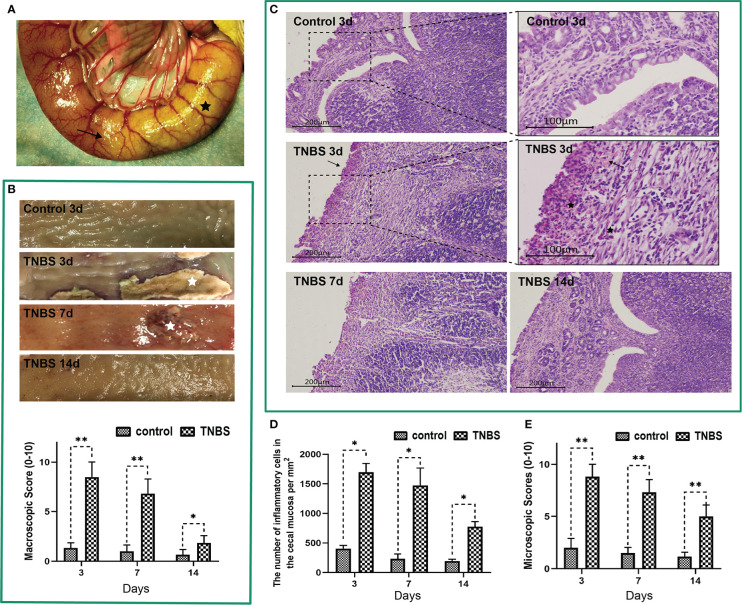
2,4,6-trinitro-benzene-sulfonic acid (TNBS)-induced cecum inflammation in rabbits (n = 6 per group). **(A)** A mixture solution of TNBS/ethanol was injected into the cecum lumen. The black star represents the cecum. The arrow shows the TNBS-injected site. **(B)** Macroscopic observations and scores of the cecum tissues. White stars represent ulcerations. **(C)** Cecum sections were stained with hematoxylin and eosin (H&E), and microscopic scores of the cecum segments were calculated. Scale bar = 200 μm. Arrows show incomplete of crypts. Representative images of TNBS-induced inflammation are shown. Black stars represent inflammatory cells (neutrophils and lymphocytes). Scale bar = 100 μm. **(D)** The number of inflammatory cells in the cecal mucosa was calculated and analyzed. **(E)** Microscopic scores of the cecum tissues. Data were expressed as mean ± SD. Statistical analysis was performed using an unpaired Mann–Whitney U-test. *P < 0.05, **P < 0.01, vs. control group.

**Figure 3:**
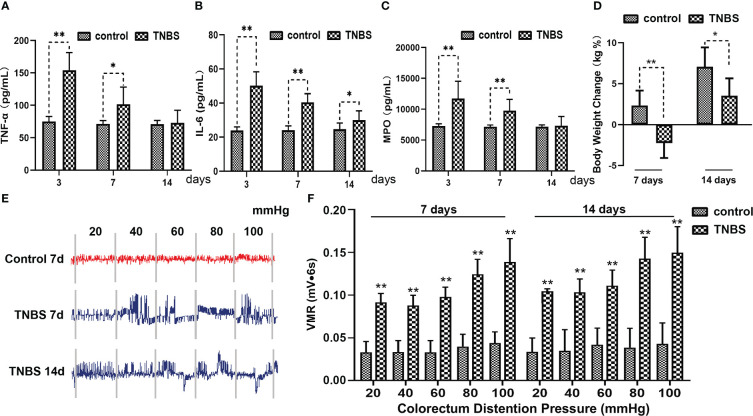
Effects of TNBS administration on the concentration of inflammatory cytokines, body weight and visceral hypersensitivity in rabbits (n = 6 per group). **(A–C)** The concentrations of tumor necrosis factor α (TNF-α), interleukin 6 (IL-6) and myeloperoxidase (MPO) in the cecum were measured using enzyme-linked immunosorbent assay (ELISA) kits. **(D)** Changes in the body weight in rabbits in the TNBS and control groups. **(E)** Representative electromyography (EMG) traces of rabbits on days 7 and 14. The pressure in the balloon was increased from 20 to 40, 60, 80, and 100 mmHg by stage (each stage lasted for 6 s). **(F)** Visceromotor responses (VMR) to colorectal distension pressure (CRD) of rabbits were analyzed from EMG traces and expressed as millivolt multiply second (mV·s). Data were expressed as mean ± SD. Statistical analysis was performed using Bonferroni’s *post hoc* test. *P < 0.05, **P < 0.01, vs. control group.

To verify PVS involvement in the process of intestinal inflammation, we took the advantage of TNBS to induce cecitis in rabbits (**Figure 2A**). As shown in **Figure 2B**, the TNBS-administrated cecum showed hemorrhage, ulcerations, adhesions and thickened walls on days 3 and 7 compared with the control group. Microscopically, TNBS induced cecum inflammation, as evidenced by distortion of crypt architecture, inflammatory cells infiltration in the submucosa, atrophy of the gland in contrast to the control group on days 3, 7, and 14 (**Figures 2C, D**). Compared with the control group, TNBS group exhibited increased (*P <* 0.05) macroscopic (**Figure 2B**) and microscopic scores (**Figure 2E**) on days 3, 7, and 14. Moreover, the concentrations of TNF-α, IL-6, and MPO in TNBS-treated cecum were greater (*P* < 0.05) than those in the control on days 3 and 7 (**Figures 3A–C**).

Compared with the control, TNBS treatment resulted in a lower body weight of rabbits on day 7 (*P =* 0.0015) and day 14 (*P* = 0.0199) (**Figure 3D**). The EMG that used to reflect the VH of rabbits is shown in **Figure 3E**. The VMRs to graded distension pressures (20–100 mmHg) were enhanced (*P* < 0.01) after cecitis was induced on days 7 and 14 compared with those in the control group (**Figure 3F**). Collectively, these findings pointed out lasting cecum inflammation, which was successfully induced by TNBS.

### The inflammation-induced proliferation of the PVS in rabbits

**Figure 4:**
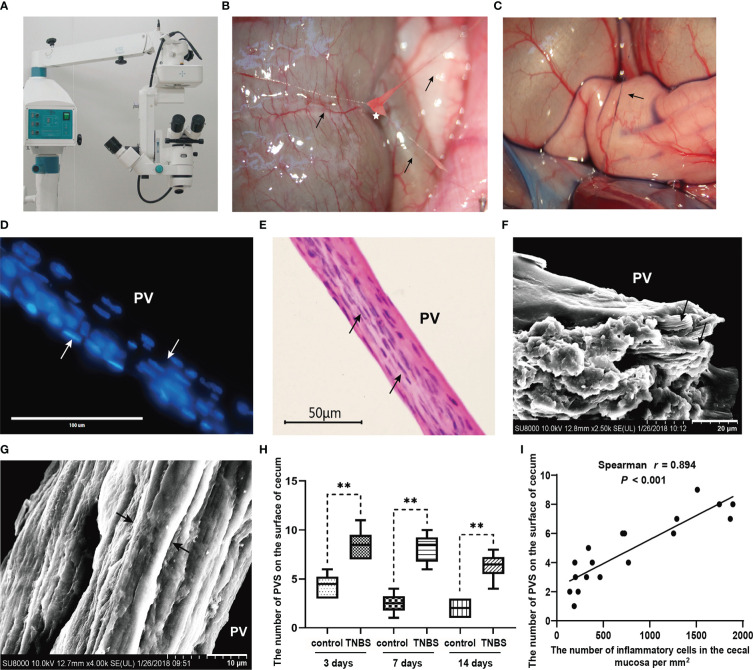
The primo vascular system (PVS) proliferated in TNBS-induced cecitis rabbits (n = 6 per group). **(A, B)** The suspected PVS samples were harvested *in vivo* under a stereomicroscope. Arrows show primo vessels (PVs). The white star represents the primo node (PN). **(C)** The suspected PVS samples were specially dyed blue by a 0.4% trypan blue solution. The arrow shows PN. **(D)** PVS sections were stained with diamidino-phenyl-indole (DAPI). Arrows show the rod-shaped nuclei of the PV. Scale bar = 100 μm. **(E)** Hematoxylin and eosin (H&E) staining of the PVS segment. Arrows show the rod-shaped nuclei of the PV. Scale bar = 50 μm. **(F)** Scanning electron microscopy (SEM) analysis of the cutting plane of the PVS. Arrows show sub-PVs. Scale bar = 20 μm. **(G)** SEM view of a typical longitudinal section of the PVS. Arrows show sub-PVs. Scale bar = 10 μm. **(H)** Changes in the number of the PVS between different groups. **(I)** Correlation (Spearman’s correlation coefficient, *r* = 0.894, *P* < 0.001) between the number of PVS on the surface of cecum and inflammatory cells in the cecal mucosa. Data were expressed as mean ± SD. Statistical analysis was performed using an unpaired Mann–Whitney U-test **(H)** and Spearman’s correlation **(I)**. **P < 0.01, vs. control group.

To elucidate the character of the PVS on the surface of inflammatory intestines, the PVS samples were captured from the surface of the cecum in TNBS-treated and control rabbits, respectively with the help of a stereomicroscope (**Figure 4A**). As shown in **Figure 4B**, the PVS was composed of PV and PN. The PVs were fine, translucent, tubular, and connected by a nodular PN. After staining with a 0.4% trypan blue solution, the PVS samples were specifically dyed blue (**Figure 4C**). The DAPI and H&E staining (**Figures 4D, E**) showed that rod-shaped nuclei of PV endothelial cells were longitudinally arranged along the PV. SEM demonstrated that the loose bundle structures of the PV consisted of multiple sub-PVs (**Figures 4F, G**). After that, the number of PVS samples were recorded and analyzed. As a result, TNBS induced an increase (*P* < 0.01) in the number of PVS at day 3, 7 and 14 compared to the control group (**Figure 4H**). Spearman’s rank correlation analysis showed that the number of PVS on the surface of the cecum was positively (*r* = 0.894, *P* < 0.001) correlated with inflammatory cells in the cecal mucosa (**Figure 4I**).

### Proteomics of the PVS on the inflammatory cecum of rabbits

**Figure 5:**
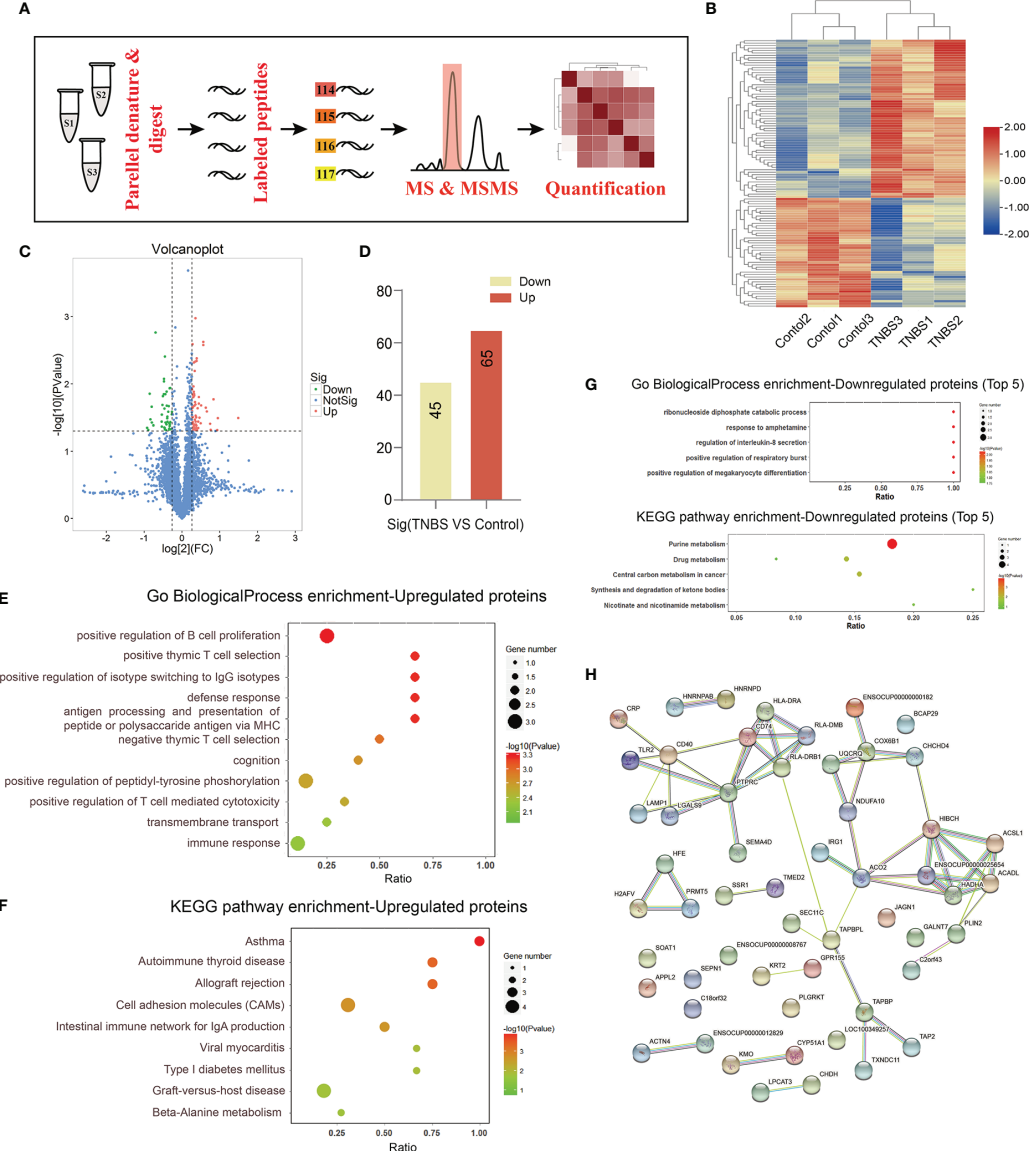
Integrated analysis of PVS by TMT-based quantitative proteomics (n = 3 per group). **(A)** The proteomic analysis of PVS is shown in the flowchart. **(B)** The heatmap shows differential expression analysis of proteins for three samples in control and TNBS groups. **(C)** Volcano plot showing screening of differentially expressed proteins. *P* < 0.05 and FC > 1.2. **(D)** The number of up- or down-regulated proteins between the control and TNBS groups. **(E)** Gene ontology (GO) biological process enrichment of up-regulated proteins. **(F)** Kyoto Encyclopedia of Genes and Genomes (KEGG) enrichment of up-regulated proteins. **(G)** GO biological process and KEGG enrichment of down-regulated proteins. **(H)** Protein–protein interaction (PPI) network of differentially expressed proteins.

To further investigate the role of increased PVs in cecitis, we collected the PVS of rabbits in the TNBS-treated group and the control group on day 7 and performed TMT-based quantitative proteomic analysis (**Figures 5A, B**). Consequently, a total of 110 differentially expressed proteins (*P* < 0.05 and FC > 1.2) were identified. Among these, 65 proteins, such as CD40, CD45, HLA-DRA1, LAMP1, and JAGN1, were upregulated, and 45 proteins, such as FGL1, protein arginase methyltransferase 5 (PRMT5), scinderin (SCIN), and nudix hydrolase 5 (NUDT5) were downregulated in the TNBS-treated group compared with the control group (**Figures 5C, D**). GO enrichment showed that the upregulated proteins were mainly enriched in the biological processes of positive regulation of B-cell proliferation, positive thymic T-cell selection, positive regulation of isotype switching to IgG isotypes, defense response, antigen processing and presentation of peptide or polysaccharide antigens *via* major histocompatibility complex (MHC). KEGG analysis showed that the differential proteins were enriched in the signaling pathways of asthma, autoimmune thyroid disease, allograft rejection, cell adhesion molecules, and the intestinal immune network for IgA production (**Figures 5E, F**). Go enrichment and KEGG analysis of downregulated proteins are shown in **Figure 5G**, and the differential proteins were mainly enriched in the signaling pathways of purine metabolism. Consistently, the protein–protein network analysis based on the STRING database yielded a similar result (**Figure 5H**). Taken together, these results indicate that the increased PVS might participate in cecitis by promoting the processes, such as inflammatory cells proliferation, antigen presentation, and cell adhesion.

**Figure 6:**
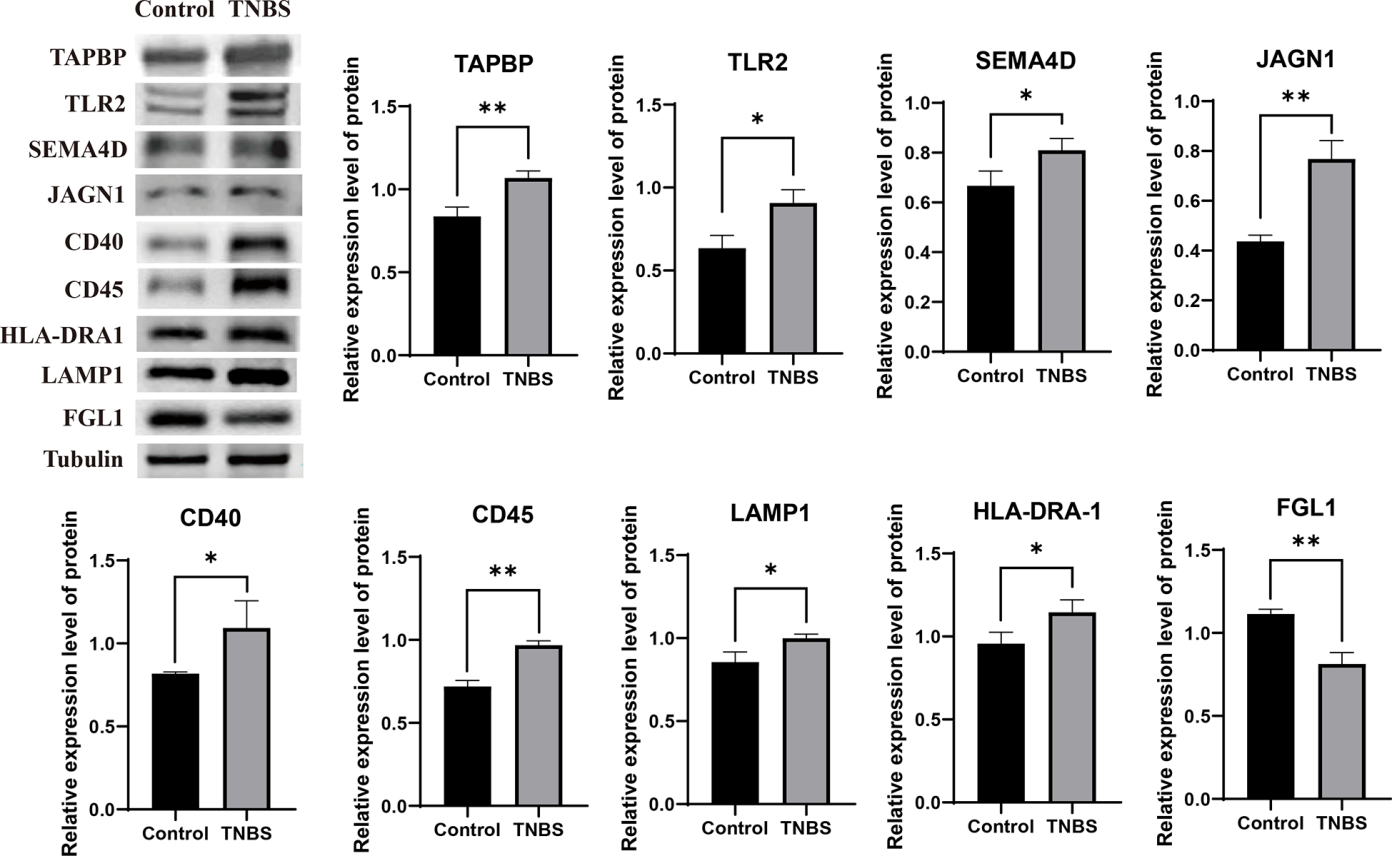
Validating the reliability of the sequencing data (n = 3 per group). Representative protein levels of TAP binding protein (TAPBP), toll-like receptor 2 (TLR2), semaphorin 4D (SEMA4D), jagunal homolog 1 (JAGN1), tumor necrosis factor receptor superfamily member 5 (CD40), anti- receptor-type tyrosine-protein phosphatase C (CD45), anti- lysosomal associated membrane protein 1 (LAMP1), HLA (RLA) class II histocompatibility antigen DR alpha chain (HLA-DRA1), and fibrinogen like 1 (FGL1) were detected in the PVS tissues by western blotting. Data were expressed as mean ± SD. Statistical analysis was performed using an unpaired Student’s t-test. *P < 0.05, **P < 0.01, vs. control group.

To validate the reliability of the sequencing data, we selected nine abundantly differentially expressed proteins for western blotting analysis. The validated proteins were consistent with the results of TMT-based quantitative proteomic sequencing. Eight proteins (TAPBP, TLR2, SEMA4D, JAGN1, CD40, CD45, LAMP1, and HLA-DRA-1) were upregulated (*P* < 0.05), whereas FGL1 was downregulated (*P* = 0.0022) (**Figure 6**).

### Modification of PVS by electroacupuncture in cecitis rabbits

**Figure 7:**
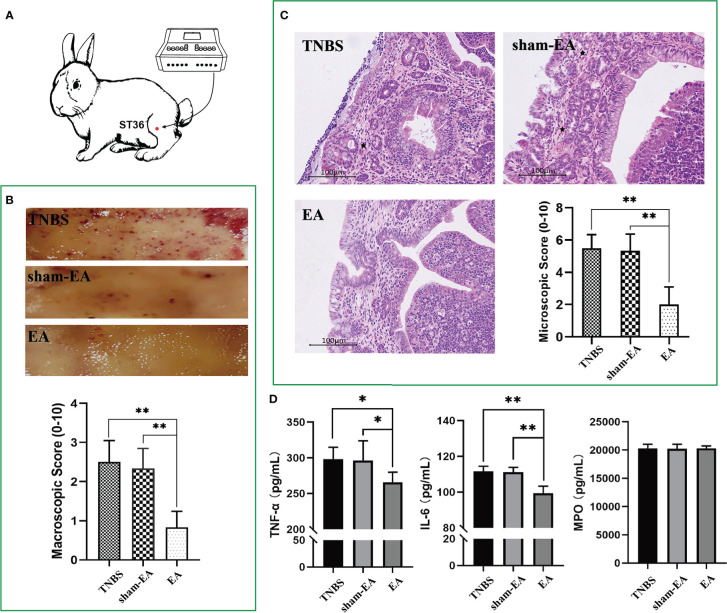
Effect of electroacupuncture (EA) on cecum inflammation and PVS proliferation (n = 6 per group). **(A)** Schematics showing the hindlimb ST36 acupoint. The TNBS-treated rabbits were given electroacupuncture stimulation on days 7, 10, 13, 16, 19, 22, and 25. **(B)** Macroscopic observation and scores of cecum tissues on day 25. **P < 0.01, vs. EA group. **(C)** Cecum sections were stained with hematoxylin and eosin (H&E), and microscopic scores of the cecum segments were calculated. Black stars represent inflammatory cells. Scale bar = 100 μm. **P < 0.01, vs. EA group. **(D)** The concentrations of tumor necrosis factor α (TNF-α), interleukin 6 (IL-6), and myeloperoxidase (MPO) in the cecum after EA stimulation were measured using ELISA kits. *P < 0.05, ** *P* < 0.01, vs. EA group. Data were expressed as mean ± SD. Statistical analysis was performed using an unpaired Mann–Whitney U-test **(B, C)** and Bonferroni’s *post hoc* test **(D)**.

**Figure 8:**
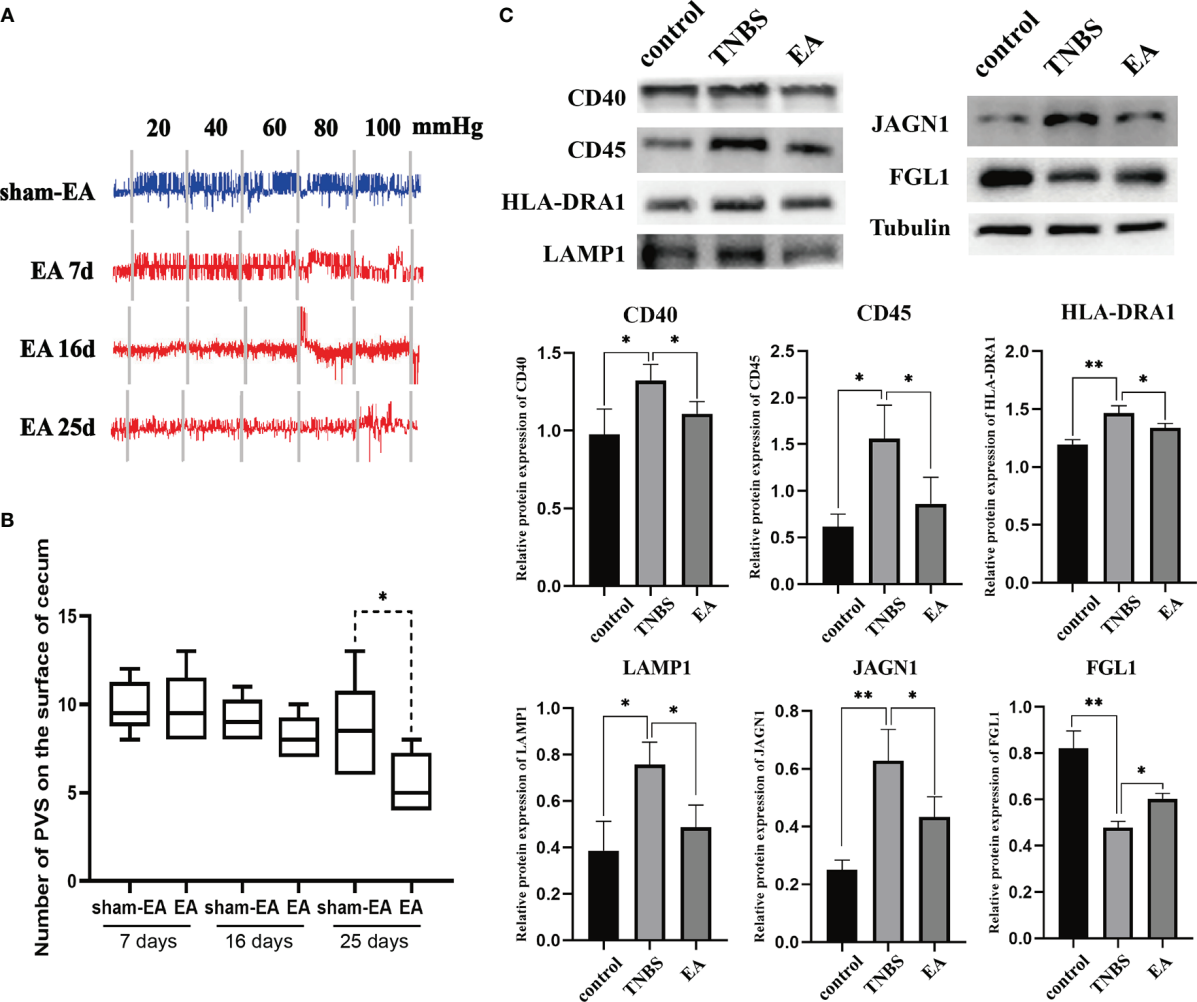
The effect of EA on PVS (n = 6 per group). **(A)** Representative EMG traces of rabbits in the Sham-EA and EA groups. **(B)** Changes in the number of the PVS tissues between the different groups. **P < 0.01, vs. EA group. **(C)** Representative protein levels of CD40, CD45, HLA-DRA1, LAMP1, JAGN1, and FGL1 were detected in the PVS tissues by western blotting. *P < 0.05, vs. TNBS group. Data were expressed as mean ± SD. Statistical analysis was performed using an unpaired Mann–Whitney U-test **(B)**, and Bonferroni’s post-hoc test **(C)**.

To clarify the association between EA and PVS in the regulation of inflammation, we performed EA stimulation at the hindlimb ST36 acupoints in TNBS-treated rabbits (**Figure 7A**). As presented in **Figure 7B**, repetitive EA stimulation relieved the hemorrhage, ulcerations, adhesions, and thickened walls induced by TNBS treatment. No difference in histopathological lesions of the cecum was observed between Sham-EA and TNBS-treated rabbits. Consistent with these results, microscopic detection shows that repetitive EA stimulation alleviated the inflammatory cell infiltration, gland hyperplasia, and goblet cell loss compared with those in Sham-EA and TNBS-treated rabbits (**Figure 7C**). In addition, the concentrations of TNF-α (*P* = 0.0342) and IL-6 (*P* < 0.001) in TNBS-treated cecum decreased after EA treatment. No difference was observed between Sham-EA and TNBS-treated rabbits (**Figure 7D**). Compared with rabbits in the Sham-EA group, EA-treated rabbits exhibited decreased (*P* < 0.05) VMR to 20–40 mmHg distension pressures on days 16 and 25 and 60–80 mmHg distension pressures on day 25 (**Figure 8A**; **Table 1**). These results suggested that repetitive EA stimulation relieves chronic cecal inflammation. Moreover, the number of PVs decreased (*P* = 0.0281) after repetitive EA stimulation compared with that in the Sham-EA group on day 25 (**Figure 8B**).

**Table 1:** Cumulative effects of repetitive electroacupuncture (EA) on visceromotor responses (VMR) to different distention pressures (n = 5 per group).

Group	Days	VMR (mV·6s)				
		20 mmHg	40 mmHg	60 mmHg	80 mmHg	100 mmHg
Sham-EA	7	0.0886 ± 0.0170	0.0896 ± 0.0096	0.0936 ± 0.0145	0.1378 ± 0.0425	0.1430 ± 0.0398
	16	0.0880 ± 0.0162	0.0950 ± 0.0130	0.0946 ± 0.0235	0.1506 ± 0.0483	0.1564 ± 0.0494
	25	0.0826 ± 0.0158	0.0906 ± 0.0180	0.1070 ± 0.0296	0.1566 ± 0.0485	0.1548 ± 0.0428
EA	7	0.0710 ± 0.0127	0.0752 ± 0.0173	0.0856 ± 0.0220	0.1474 ± 0.0436	0.1452 ± 0.0386
	16	0.0552 ± 0.0116*	0.0612 ± 0.0239*	0.0644 ± 0.0099	0.1252 ± 0.0315	0.1366 ± 0.0411
	25	0.0530 ± 0.0084*	0.0534 ± 0.0084*	0.0512 ± 0.0158*	0.0862 ± 0.0137*	0.1208 ± 0.0196

*P < 0.05, vs. Sham-EA group. Data were expressed as mean ± SD. Statistical analysis was performed using Bonferroni’s post-hoc test.

Given that TNBS induces an increase in inflammation-related proteins (CD40, CD45, HLA-DRA1, LAMP1, and JAGN1) in the PVS, as shown by proteomic analysis, we determined whether these differential proteins in the PVS were also regulated by EA. Consequently, the expression of CD40, CD45, HLA-DRA1, LAMP1, and JAGN1 were increased (*P* < 0.05) in the PVS on the inflammatory cecum of rabbits, and reversed by EA treatment. Additionally, TNBS induced a decrease (*P* < 0.01) in the expression of FGL1 and a reversed (*P* = 0.0466) by EA stimulation (**Figure 8C**). Taken together, these results revealed that EA suppressed the proliferation of PVS in the inflammatory cecum of rabbits and downregulated proinflammatory proteins in it.

## Discussion

Acupuncture has been used for a long time in China. From 60’s of the last century, Chinese physicians modified acupuncture by combining it with some modern techniques (such as electricity and electromagnetism), and started its extensive research and clinical practice. Acupuncture has been widely used to prevent pain during and after operations and to treat acute and chronic diseases, such as knee osteoarthritis (40), chronic musculoskeletal pain (41), chronic severe functional constipation (42), inflammatory bowel disease (22, 43), and intervertebral disk disease (44, 45) in humans and animals. However, its peripheral regulating mechanisms have not yet been clarified. According to traditional Chinese medicine theory, there is a complex network system consisting of 12 main channels with branches (collaterals), which is called the “meridian” system. The meridian system is thought to be a channel for the circulation of “Qi” and “blood”. Acupuncture takes effect by influencing the balance of “Qi” and “blood” in the system. The meridian is the core theory that guides the diagnosis and application of traditional Chinese medicine in a way completely different from Western medicine. However, the anatomical foundation of the meridian is a missing piece of the map to fully understand traditional Chinese medicine and is thought to be able to bridge the gap between Chinese and Western medicines. Over the past 60 years, the physical reality of acupuncture meridians has been confirmed by electroconductivity (46, 47), hydraulic conductance (48), acoustic wave propagation (49, 50), detectible light and heat delivery (49, 51, 52), radioisotope tracing pathway (53, 54), and the propagated sensation along the channel. There are different hypotheses that are used to explain it, including nervous (55–57), humoral (58), energic (59), and fascia (60) theories. However, there is no known anatomical foundation for the meridians.

Kim proposed that the PVS is a new net-like system in the body, consisting of the “primo node” and the “primo vessel,” claimed to be acupuncture meridians. Over the past 20 years, PVS researchers have applied multiple histological staining methods and modern experimental techniques (radioisotope tracing, fluorescent nanoparticles, quantum dot, immunoaffinity chromatography, and electron microscopy), and have verified that PVS exists in the intra-vascular and intra-lymphatic vessels, on the surface of internal organs and in the central nerve system (8–11). Han et al. (29) intratesticularly injected a chromium hematoxylin and fluorescent nanoparticle solution and found that the dye and nanoparticles occurred in the PVS on the surface of the greater omentum, small intestine, and urinary bladder, indicating that the PVS may exist in the parenchyma of organs, and links to others. The PVS in the skin is believed to be an important structure because of the role of its bonds between external stimulation and internal organs. Kim et al. (61) injected a vascular casting material (polymer Mercox) into acupuncture points as a tracer, partially macerated the whole body with a potassium-hydroperoxide solution, and found that the PVS was visualized along the routes of the meridian system under a stereomicroscope. Kwon et al. (10) characterized the PVS with immunohistochemistry, and found that its nodes and ducts were covered by a layer of EMP-3-positive spindle-shaped epithelium with, below, a layer of vWF-positive but CD31- or LYVE-1-negative endothelium. Cho et al. (62) applied an intracellular recording technique to measure the action potentials of the PVS and found that its full widths at half the maximum (FWHMs) of the action potentials were tens of milliseconds, which were significantly different from those of a neuron, a skeletal muscle cell, and a cardiac muscle cell. Cho et al. (63) introduced a mathematical analysis method, a normalized Fourier transform that displays the sine and cosine components separately, to compare the action potentials of PVs with those of smooth muscles. They found that PVs generated two types of action potential pulses that differed from those of smooth muscles. These studies indicate that the PVS is a unique system that differs completely from blood vascular and lymphatic vessels and neurological tissues; its distributional and bioelectrical properties coincide with those of the meridian system.

The functions of the PVS have been the focus of attention. Kwon et al. (10) reported that the resident cells in the PVS consisted of a variety of active cells. Among them, mast cells, eosinophils, neutrophils, histocytes, lymphatic cells, chromaffin cells and immature microcells accounted for 20.0 ± 4.3%, 16.4 ± 5.6%, 5.0 ± 0.1%, 53.6 ± 2.7%, 1.0 ± 1.0%, 0.3 ± 0.3%, and 2.9 ± 0.1%, respectively. Lee et al. (64) reported that the number of the immature microcells (p-microcells, a stem cell) with Sca-1(+) Lin (–) CD45 (-) in the PVS was approximately 100-fold greater than very small embryonic-like stem cells in the bone marrow, and injection of the p-microcells into mice partially repaired ischemic brain damage. According to PVS inclusion properties, the effects of PVS may be involved in physiological and pathological processes of inflammation, pain, immunity, damage repair, and tissue regeneration. In addition, the new PVS formed at a higher density in or around the tumor were found to participate in cancer cell transport, and to facilitate the dissemination and colonization of cancer cells at secondary sites (65, 66). However, how acupuncture affects the role of the PVS remains unknown.

Research progress in PVS functions is restricted by the lack of a sufficient number of PVS samples to be analyzed due to its extremely tiny structure, and by the lack of proper pathological models to display its inclusion change. The identification of the PVS has been performed by taking advantage of its natural anatomical structure, and the PV is known to have unique characteristics according to the reports: (1) multiple sub-PVs in a single PV, and the inter-sub-PV space filled with fibrous, amorphous substances and (2) rod-shaped nuclei of sub-PV endothelial cells. In the present study, tiny thread-like structures were visualized with trypan blue staining *in vivo*, and collected under an optical stereomicroscope. The harvested samples with rod-shaped nuclei of endothelial cells were identified as PVs, which is similar to previous reports. TNBS is commonly used to induce intestinal inflammation and to investigate the mechanisms underlying IBD and VH. Rabbits have a blind sac-like cecum with 10 times the capacity of their stomach and commonly suffer from cecitis. Considering the sufficient quantity of tiny PVS on the cecum to be collected, local inflammation and hyperalgesia were induced by injecting TNBS into the lumen of the cecal terminal end of the rabbits in this study. This model may have several advantages for studying acupuncture-mediated PVS functions: inflammation is limited to the local site of the cecal end, and hyperalgesia exists for a long time; the PVS on the surface of the intestine is easy to observe or sample. More importantly, acupuncture is effective in relieving the intestinal inflammation and hyperalgesia. Applying this model, we found that increased PVs on the surface of the cecum were accompanied by inflammation and hyperalgesia, which were positively correlated with the inflammatory cells in the cecal mucosa. These studies indicated that the PVS might be involved in the initiation and development of local enteritis.

TMT labeling has become a versatile technique in relative protein quantification with high throughput, reproducibility, and sensitivity, and is widely used for profiling and quantifying differentially expressed proteins. In this study, we used TMT labeling and liquid chromatography-mass spectrometry/mass spectrometry to characterize protein alterations in the PVS on the surface of the cecum with inflammation. Proteomic analysis showed that multiple biological progresses, including B-cell proliferation, thymic T-cell selection, antigen processing and presentation *via* MHC were positively regulated in the proliferated PVS. A total of 110 differentially expressed proteins (65 upregulated proteins and 45 downregulated proteins) were identified. Among these, the expression levels of inflammation-associated proteins, including CD40, CD45, HLA-DRA1, LAMP1, and JAGN1, of the PVS, were upregulated in TNBS-treated rabbits. Importantly, these upregulated PVS proteins in the inflammatory state were reversed by repetitive EA stimulation. CD40 has been reported to initiate the intestinal mucosal inflammation in IBD patients by interacting with CD154 (67). Previous studies have reported that CD45 is required for T-cell activation through the antigen receptor, and CD45^+^ cells were found to profoundly infiltrate the intestinal mucosa of IBD mice (68). HLA-DRA1, as an antigen presentation protein, was found upregulated in IBD patients (69). LAMP1 has been reported to induce the activation of the NLRP3 inflammasome, which promotes the maturation and secretion of the pro-inflammatory cytokines in inflamed intestines (70–73). According to a previous study, JAGN1 has been identified as indispensable for the biological function of neutrophils (74), which accumulate in the inflamed mucosa of IBD. In addition, the expression level of FGL1 was downregulated in TNBS-treated rabbits and reversed by repetitive EA stimulation. FGL1 is a major inhibitory ligand for LAG-3. Ablation of the FGL1-LAG-3 interaction in mice promotes T-cell immunity, which is widely considered the main pathogenesis of IBD (75, 76). Altogether, these findings indicate that EA stimulation displayed a significant anti-inflammatory effect, probably by suppressing the proliferation—or regulating the inflammation-related protein expression—of the PVS.

In summary, the PVS, a net-work system independent of the arteriovenous and lymphatic systems, was claimed to be a presentative of the acupuncture meridian. Research on the relationship between PVS and acupuncture effects is conducive to elucidating the entity of the meridian. Our preliminary results demonstrated that the PVS was involved in the development of inflammation, and the EA modified its participation in this procedure. Although the necessity of the PVS-mediated acupuncture effect needs to be verified, our study provides a new avenue to further explore the peripheral mechanism by which EA relieves inflammation.

## Data availability statement

The datasets presented in this study can be found in online repositories. The names of the repository/repositories and accession number(s) can be found below: http://www.proteomexchange.org/, PXD034280.

## Ethics statement

The animal study was reviewed and approved by Institutional Animal Care and Use Committee of Huazhong Agricultural University (HZAURAB-2022-0002).

## Author contributions

Conceptualization, YD. Methodology, SN and JW. Software, QL and RY. Formal Analysis, XW, NM, and JZ. Data Curation, SN, JW and QL. Writing -Original Draft Preparation, SN. Writing - Review Editing, YD and MD. Project Administration, YD. Funding Acquisition, YD and MD. All authors contributed to the article and approved the submitted version.
