# Supplementary material for: The involvement of the primo vascular system in local enteritis and its modification by electroacupuncture
Source: Front Immunol. 2023 Jan 11;13:1072996. doi: 10.3389/fimmu.2022.1072996 (PMC9874324; doi:10.3389/fimmu.2022.1072996)
Supplement: Supplementary file 2 [file Table_1.docx]

**Table S1 Macroscopic scoring criteria**

| *Feature* | *Score* |
| --- | --- |
| *Ulceration* |  |
| normal appearance  focal hyperaemia, no ulcers  ulceration without hyperaemia or bowel wall thickening  ulceration with inflammation at one site  ≥ two sites of ulceration and inflammation  major sites of damage extending >1 cm along the length of cecum  damage extended to >2 cm along the length of the cecum, increase the score by one for each additional cm of damage | 0  1  2  3  4  5  6-10 |
|  | plus |
| *Adhesions* |  |
| no adhesions to surrounding tissues  minor adhesions  major adhesions | 0  1  2 |
| *Diarrhoea* |  |
| absence of  presence of | 0  1 |
|  | Total score |

**Table S2 Microscopic scoring criteria**

| *Feature* | *Score* |
| --- | --- |
| *Crypt architecture* |  |
| normal  < 30% reduction of crypt length  > 30% reduction of crypt length and loss of goblet cells  complete loss of crypts | 0  1  2  3 |
| *Inflammatory cell infiltration* |  |
| no infiltration  infiltration of inflammatory  extending into the submucosa  transmural infiltration | 0  1  2  3 |
| *Ulceration* |  |
| intact epithelium  ulceration limited to lamina propria  extending to submucosa  transmural ulceration | 0  1  2  3 |

**Table S3 The readout of proteomic (differentially expressed proteins)**

| **Accession ID** | **Protein** | **Control1** | **Control2** | **Control3** | **TNBS1** | **TNBS2** | **TNBS3** |
| --- | --- | --- | --- | --- | --- | --- | --- |
| G1SWH6 | CHDH | 1540.1 | 1003 | 1270.3 | 1862.8 | 2284.9 | 1853.4 |
| G1SCU2 |  | 219.6 | 151.4 | 157 | 241.7 | 286.8 | 287.2 |
| G1TA26 | SLC15A3 | 421.6 | 307 | 364.4 | 450.6 | 474.9 | 546.8 |
| G1SS57 | SCIN | 5553.4 | 4162.4 | 6070.3 | 3185.5 | 3313.4 | 2562.4 |
| G1TZR2 | C18orf32 | 1676.6 | 1274.6 | 1439.5 | 1945.5 | 1785.2 | 2179.4 |
| G1TX81 | LGALS9 | 6191.7 | 4856.4 | 5406.9 | 6721.5 | 6439.8 | 7339 |
| G1T2U1 | PPAT | 708.1 | 557.8 | 625.4 | 457 | 534.5 | 391.3 |
| G1TI70 | NPL | 993.7 | 785.4 | 953.4 | 640.3 | 597.5 | 296.5 |
| G1SF95 | HNRNPD | 5325.7 | 4214.7 | 4868.2 | 6035.5 | 5744.7 | 7143.3 |
| G1TPN3 | HNRNPAB | 16503.2 | 13068.3 | 13998.9 | 18060.2 | 17414.4 | 20915 |
| A7X8X3 | HPRT | 11843.1 | 9421.2 | 10810 | 7807.5 | 9020 | 6486.4 |
| G1TEU8 | TBCC | 343 | 278 | 334.2 | 245.9 | 250.8 | 265.5 |
| G1U3T2 | MAP7D1 | 1599.5 | 1301.3 | 1522.9 | 1257.8 | 1122.3 | 913.9 |
| G1T2X0 | PLIN2 | 741.2 | 606.1 | 841 | 1091.7 | 1053.8 | 1576.4 |
| G1SDV3 | BDH2 | 969.6 | 797.9 | 914.8 | 721 | 708.6 | 671.7 |
| G1SH52 | RCSD1 | 2000.5 | 1647.8 | 2183 | 1465.8 | 1110.3 | 1444.4 |
| G1TBV6 | ATP13A3 | 233.7 | 193.1 | 228.8 | 248 | 291.4 | 268.3 |
| G1TUC8 | ACTN4 | 28127.4 | 23363.2 | 24643 | 31067.8 | 31428.2 | 31415.5 |
| G1SQ01 | LOC100346892 | 10440.9 | 8681 | 8999.8 | 7171 | 7940.5 | 6036.9 |
| G1SMC0 | CRP | 1050.8 | 876.4 | 1228 | 705.2 | 713.2 | 395.4 |
| G1SP30 | FKBP5 | 7813 | 6519.8 | 6839.3 | 5431.1 | 5265.7 | 4096.6 |
| G1TBJ1 | ZNF879 | 848.8 | 708.6 | 1087.8 | 593.1 | 517.8 | 285 |
| G1SN35 | APPL2 | 238 | 198.8 | 209 | 251.2 | 266.6 | 269.9 |
| G1SR56 | DCUN1D5 | 1476.2 | 1233.9 | 953.8 | 837.4 | 744.4 | 588.5 |
| G1SP32 | HIBCH | 7863.7 | 6610.5 | 7266.1 | 8774.5 | 8301.5 | 9431.5 |
| G1TP80 |  | 646.4 | 545 | 585.3 | 473.5 | 330.9 | 153.9 |
| G1SFG6 | GPI | 23744.7 | 20081.3 | 20308.1 | 14752 | 18534.7 | 14081.6 |
| G1SFF5 | SPTLC2 | 3793.4 | 3243.2 | 3382.9 | 4145.3 | 4316.2 | 4399 |
| G1T8D5 |  | 760.7 | 652 | 825.2 | 917.3 | 1213.7 | 1022.9 |
| G1SRD2 | ACADL | 7234.7 | 6203.3 | 7140.4 | 8885.7 | 8722.3 | 9257.2 |
| G1TF72 | CHCHD4 | 1605.6 | 1379.4 | 1347.1 | 1601 | 1837.2 | 1834.7 |
| G1SUQ0 | SOAT1 | 3465 | 2980.9 | 3639.9 | 4148.9 | 4058.4 | 4722.9 |
| G1TA86 | LDAH | 769.6 | 663.4 | 762.8 | 888.1 | 873.3 | 989.7 |
| G1TCS5 | TXNDC11 | 161.8 | 139.5 | 142.1 | 172.7 | 176.3 | 189.2 |
| G1T7D7 | HFE | 1604.9 | 1391.8 | 1593.4 | 1864.5 | 1731.7 | 1918 |
| G1T774 | NHSL2 | 326.2 | 283 | 328.7 | 244.1 | 247.7 | 215.3 |
| G1SPS9 | APBB1IP | 7483.1 | 6501.9 | 6839.8 | 5350.8 | 6071 | 4563.7 |
| G1T2D8 | PTPRC | 33232.1 | 29066.5 | 30230.1 | 33922.9 | 39856.8 | 38684.4 |
| G1U903 | LRRC8D | 206.8 | 182.3 | 194.8 | 230.4 | 234.9 | 259.4 |
| G1U3F9 | SH3BP4 | 1845.4 | 1638 | 1614.8 | 1395.6 | 1361.2 | 1354.9 |
| G1SE16 | LEMD3 | 610.3 | 544.2 | 615.3 | 678.5 | 698.7 | 761.4 |
| G1SWH9 | SSR1 | 3287.1 | 2932.4 | 3081.3 | 3772 | 3671.1 | 4192.3 |
| G1TXW3 | RLA-DMB | 220.6 | 196.9 | 202.8 | 290.6 | 438.5 | 313.3 |
| G1T235 | PSMB6 | 4044.4 | 3610.2 | 3833.2 | 3072.2 | 3156 | 2086.2 |
| G1SKK3 | CD74 | 4181.9 | 3734.5 | 3168.3 | 5344.1 | 7666.4 | 6793.6 |
| G1TY95 |  | 110 | 98.3 | 116.7 | 92.2 | 90.7 | 76.7 |
| G1T0K4 | GALNT7 | 1358.7 | 1217.6 | 1333.5 | 1485.9 | 1868.1 | 1720.7 |
| G1T330 | WARS2 | 1312.2 | 1176.9 | 1350.2 | 1449.3 | 1507.9 | 1682.8 |
| G1ST02 | QPCT | 269.9 | 243.2 | 307.3 | 217.8 | 220.5 | 210.5 |
| G1SEI1 | CLASP2 | 1181.8 | 1067.3 | 1142.1 | 888.9 | 974.6 | 837.9 |
| G1T1N2 | CAMK1D | 2235.2 | 2018.7 | 1933.8 | 1472.3 | 1691.5 | 1117.9 |
| G1SUG3 | PLGRKT | 3382.4 | 3056.8 | 3076.6 | 4067.1 | 4340.7 | 3665.7 |
| Q7YQK4 | SLC7A5 | 5344.5 | 4843.5 | 4368 | 3946.1 | 3647.7 | 3419.4 |
| G1TA72 | HS1BP3 | 402 | 364.8 | 357.8 | 295.3 | 296.2 | 237.8 |
| G1SRI9 | MAN1A1 | 4106.5 | 3727 | 3681.4 | 4421.2 | 4486 | 5221 |
| G1SWZ1 | NUDT5 | 359.2 | 326 | 363.8 | 280.5 | 315.2 | 277.7 |
| G1SXX5 | HADHA | 33633.2 | 30656.4 | 32859.6 | 36695.9 | 38559.6 | 44412.7 |
| G1SXI9 | COX6B1 | 548.7 | 503.8 | 553.7 | 604.5 | 677.2 | 749.1 |
| G1SFM2 | BRI3BP | 305.9 | 281.9 | 306 | 343.5 | 403.1 | 344.3 |
| G1SM08 | CYP51A1 | 352.5 | 325.3 | 387.4 | 401.3 | 506.3 | 479.9 |
| G1SZQ1 | MCM5 | 1219.5 | 1125.8 | 1082.6 | 914.2 | 1027.3 | 821.3 |
| G1T7U7 | NAAA | 5099.5 | 4714.3 | 4676.4 | 5711.9 | 5839.4 | 6542.8 |
| G1U724 | SAE1 | 4255.5 | 3942.4 | 3523.5 | 2959.1 | 3327.5 | 2650.2 |
| P11974 | PKM | 41128.3 | 38152.5 | 40767.9 | 27195.4 | 30575.6 | 35493.9 |
| G1SF37 | MFSD1 | 402.1 | 373.5 | 357.7 | 510.2 | 442.9 | 553.5 |
| G1SWZ7 | CDC123 | 129.9 | 120.9 | 120.2 | 108 | 104.7 | 81.1 |
| G1SWR7 | SEC11C | 1905.4 | 1782.8 | 1800.8 | 2294.5 | 2436.6 | 2297.3 |
| G1TRZ2 | LAMP1 | 4967.9 | 4672.9 | 4746.2 | 5834.7 | 5493.1 | 6387.6 |
| G1T5S9 | UQCRQ | 3484.5 | 3286.4 | 3289.1 | 3952.8 | 4020.1 | 4232.8 |
| G1U0U5 | HSPA4L | 4937.7 | 4657.7 | 5088.8 | 3745.7 | 3798.8 | 3008.6 |
| G1TYI8 | SELENON | 201.2 | 190 | 221.1 | 262.6 | 236.9 | 236.7 |
| G1SYR2 | PTCD3 | 1130.2 | 1073.5 | 1159.4 | 1322.4 | 1397.2 | 1465.8 |
| G1SQN2 | SH3KBP1 | 4702.2 | 4480.9 | 4506.9 | 4088.6 | 3913.9 | 3357.3 |
| G1SVL5 | LOC100349257 | 530.7 | 506.2 | 531.4 | 624.3 | 796.3 | 802.9 |
| G1SUH8 | KRT2 | 1858.2 | 1784.8 | 1702.5 | 2223.9 | 2582.5 | 2051.7 |
| G1SJR4 | TMED2 | 7642 | 7371.9 | 7886.8 | 9682.5 | 8486.4 | 11015 |
| G1TZQ6 | NDUFA10 | 10484.9 | 10150.7 | 9763.7 | 11795.5 | 12286.6 | 13568.8 |
| G1SNM8 | JAGN1 | 528.7 | 512 | 496.8 | 634.4 | 572.1 | 640 |
| G1U891 | LOC100347496 | 1958.7 | 1899.7 | 2007 | 2356.2 | 2174.9 | 2696.8 |
| G1TUX2 | ACO2 | 24824.3 | 24087.9 | 25681.3 | 30444.6 | 29253.8 | 37757.1 |
| G1SFZ5 | GCFC2 | 109.2 | 106.1 | 111.5 | 153 | 153.6 | 176.3 |
| G1SFC3 | RLA-DRB1 | 2438.2 | 2374.5 | 2925.8 | 4846.3 | 9806.1 | 7075.2 |
| G1T821 | FGL1 | 3835.6 | 3737.8 | 4362.1 | 3218.2 | 2694.7 | 2505.6 |
| Q30847 | RLA-DR-alpha | 4811 | 4704 | 4767.5 | 7594.3 | 11935.3 | 8060 |
| G1SGM2 | PRMT5 | 5966.6 | 5857.8 | 6036.7 | 5133.5 | 5135.5 | 3798.4 |
| G1TPZ4 |  | 10595.5 | 10464.1 | 9912.1 | 12688.3 | 11521.4 | 14400.5 |
| G1T887 |  | 1131.4 | 1117.7 | 1168.2 | 1347.8 | 1654 | 1643.7 |
| Q95MN1 | NCF4 | 8718.9 | 8620.9 | 8424.6 | 6598.5 | 6872.7 | 5019.6 |
| G1SIY4 | KIAA0513 | 2221.7 | 2207.3 | 2037.4 | 1551.3 | 1719.5 | 1457.3 |
| G1SJJ7 | TLR2 | 1818.6 | 1828.6 | 1782.9 | 2137.1 | 2395.2 | 2398.8 |
| G1SDC9 | ARHGAP15 | 954.4 | 961.2 | 918.4 | 697.6 | 801.6 | 554.8 |
| G1SPC6 | CD40 | 681.3 | 688.4 | 783.3 | 940.2 | 906.9 | 971 |
| G1TNZ8 | TAP2 | 4740.5 | 4816.5 | 4013.9 | 6375.7 | 7018.5 | 6664.3 |
| G1SUN1 | ARL6IP5 | 6467.7 | 6598.2 | 6293.7 | 7752.6 | 7894.6 | 8742.1 |
| G1SSN5 | ATXN10 | 906 | 924.5 | 895.4 | 748.3 | 769.9 | 565.8 |
| G1SK76 | GPR155 | 1234 | 1282 | 1282.6 | 1573.4 | 1841.4 | 1441.6 |
| P14461 | FGA | 681.7 | 741.1 | 715.2 | 467.7 | 589.8 | 480.5 |
| G1T4F3 | SEMA4D | 1107.3 | 1212.1 | 1083.9 | 1296 | 1424.2 | 1453.4 |
| Q08863 |  | 111.9 | 123.7 | 115.8 | 77.9 | 76.1 | 62.2 |
| G1SUL5 | TAPBPL | 482.8 | 535.8 | 477.9 | 617.6 | 645.6 | 793.7 |
| G1TFX2 | LOC100328621 | 408 | 453.1 | 498.9 | 365.7 | 329.4 | 388.4 |
| G1T7G3 | LPCAT3 | 3877.2 | 4306.6 | 3731.7 | 5273.6 | 4876.3 | 4728.2 |
| G1SZK1 | TAPBP | 3611.8 | 4035.8 | 3323.6 | 4664.4 | 4717.9 | 5530.6 |
| G1T5D3 | NAMPT | 2225.4 | 2489.8 | 2068.9 | 1917.1 | 1924.1 | 1651.3 |
| G1TAL0 | ARHGAP12 | 725.7 | 818.3 | 783.3 | 639.1 | 584.2 | 383.9 |
| G1SPB6 | ACSL1 | 1749.8 | 2094 | 2233.5 | 2557 | 2532.9 | 2692.5 |
| G1STL6 | KMO | 2058.7 | 2547.4 | 2023.7 | 2987.6 | 2590.2 | 2950.7 |
| G1U747 |  | 723.3 | 922.8 | 1017.5 | 506.4 | 549.5 | 417.7 |
| G1TRS2 | ACOD1 | 4938.5 | 6522.6 | 5314.3 | 8238.6 | 9748.2 | 14092.9 |
| G1TRK9 |  | 1436.9 | 2283.1 | 2099.8 | 1267.7 | 1160.1 | 1019.3 |
